# The A. D. A. Meeting

**Published:** 1887-06

**Authors:** 


### Editorial.

### THE A. D. A. MEETING.

It was the wish of many good members of the American Dental Association that a meeting should not be held this year. Others were fully persuaded that to suspend the meetings for a year would result in the weakening of the influence of the society upon dentists, and might be interpreted as an abdication of its claim to be the representative National Association, for it would be a confession of vacillation and lack of vigor. Besides, there was a serious doubt if the officers could, under the constitution, refuse or neglect to call the meeting. That this question might be decided before final action was taken, we determined to do two things: first, to enter a protest, that it might not be claimed that there was no objection to passing the meeting of 1887; and second, to call the attention of the officers to the provisions of the constitution, that illegal action might not unwittingly be taken. A letter was therefore addressed to the secretary, signed by Prof. Abbott and the editor of this journal, in which the point was raised, which was really one of law, although it was called a point of order to bring it within the province of the president to decide. It should be distinctly understood that this action was not taken in a spirit of factious opposition, but that a gross error might not be committed, and the society thus placed in a very embarrassing position. The signers of the subjoined letter will be as ready to accept the decision of the proper authorities as will any others of the members.

*George H. Cushing. Secretary of the American Dental Association:*

We, the undersigned, desire to file with you for transmission to the president and officers of the Association, our protest against the abandonment of the regular annual meeting of the Association for this year, and to raise the following point or points of order, which we desire should be laid before the board, and which it will be necessary to overrule before such action can legally be taken.

POINT OF ORDER.

That under the constitution the Executive Committee has no power to declare that a meeting shall not be held. Article four provides as follows:

The regular meetings of the Association shall be held annually, and commence on the first Tuesday in August. The place of meeting shall be determined each year by vote of the Association.

An amendment to this article was adopted in 1882, as follows:

Sec. 2. The officers may, for extraordinary reasons, change the time and place of meetings upon the written consent of ten (10) of the fifteen (15) officers.

We submit that power to change the time and place of meeting was delegated to the officers for a specific reason, and that it in no sense conveys authority to abandon the meetings altogether. If this were the case, the officers would have it in their power to destroy the organization by refusing to call the regular annual meetings at any time.

In 1881 the time was changed to allow those who desired to attend the meeting of the International Medical Congress in London to be present at both. This was done without any authority, but it stood because of there being no objection raised. At the meeting so convened, the amendment now known as Sec. 2 of Art. IV of the constitution was offered to meet such emergencies, and at the next regular meeting it was adopted. But we submit that this never contemplated the total abandonment of any meeting. The time of the meeting of 1881 was changed to *allow* members to attend both meetings, whereas it is now, in effect, proposed to intermit the meeting of the Association to *prevent* the attendance upon both.

The neglect and refusal of the officers to call the meeting of the Association could not be excused under Sec. 2 of Art. IV, because such action could not be considered a change of time within the meaning of the amendment. According to the constitution a meeting *must* be held during the year 1887. The officers have no discretion in the matter. The time cannot be changed to 1888, for the meeting so held would not, within the meaning of the constitution, be that of 1887, and could not be construed as a mere change of the time for holding such meeting. It would necessarily become the meeting of 1888.

The officers may change the time and place for holding any annual meeting, but they cannot legally or without gross neglect of a plainly prescribed duty refuse to call the regular annual meeting *for this year.* They must either allow the time and place fixed by the Association at Niagara last year to stand, or they must change either or both within the plain meaning of the constitution. They cannot utterly refuse to call the regular annual meeting for the year 1887. Respectfully submitted,

W. C. Barrett.
Frank Abbott.

This letter was placed in the hands of the President, who gave it the consideration which it demanded, and in due time announced his decision as follows :

Chicago, Ill., May 6th, 1887.

*Dr. Geo. H. Cushing, Recording Secretary, American Dental Association :*

Dear Sir—Your letter of April 30th, accompanied with a copy of a communication from Drs. W. C. Barrett and Frank Abbott, making a protest against a proposed action of the officers of the Association looking to the postponement of the regular annual meeting of the American Dental Association of the present year until 1888, and raising what they term a "point of order" as to the power of the officers under the constitution to do so, also your letter of more recent date with a statement of the vote upon the question submitted to the officers of the Association, is received.

As the question raised had not occurred to me before, and the existing circumstances are of more than ordinary importance, I have thought it best to take such time to consider the subject as would enable me to reach a correct conclusion as to the power of the officers to change the time and place of the Association's annual meeting; hence the delay in my reply.

It should be noticed that the gentlemen do not question the power of the officers to change the time and place of meeting, provided the time is fixed within the year 1887.

The authority that the officers have (if such authority exists) to postpone the next annual meeting to 1888, is found in Section two (2) of Article four (4) of the constitution. So much of the article as in any way governs the question, and to which reference is made by the protestants, reads as follows:

"Time of Meetings.—The regular meetings of the Association shall be held annually, and commence on the first Tuesday in August. The place of meeting shall be determined each year by vote of the Association."

"Sec. 2.—The officers may, for extraordinary reasons, change the time and place of meeting upon the written consent of ten (10) of the fifteen (15) officers."

As Drs. Barrett and Abbott are not officers of the Association, and have no right to vote upon the question at issue, or upon an appeal from any decision made, I could not see clearly how they could raise a point of order.* The question, it seemed to me (for the time being at least), was a matter that rested entirely with the officers of the Association.

The point they raise is valuable, however, as it has suggested a more thoughtful consideration of the question as to whether the officers have the power to omit a regular annual meeting of the Association, for if the regular meeting for this year is postponed until 1888, the meeting of 1887 or that of 1888 would, of necessity, have to be omitted.

Upon a careful reading of the article referred to, I became satisfied that an honest construction of its meaning forbade the officers postponing the meeting until next year. But before giving this as my decision, I felt that I should take counsel with some one in whose opinion the Association could rest with assured confidence.

With this idea in view, I placed the constitution of the Association and the protests of Drs. Barrett and Abbott in the hands of Ex-United States Senator Lyman Trumbull, and asked him to give me his written opinion upon the entire subject, which will be found in the following copy of a letter received from him:

"Chicago, May 4th, 1887.

"*Dr. W. W. Alport, President American Dental Association, Argyle Building, City.*

"Dear Sir—The authority of the officers of the American Dental Association to dispense with the annual meeting is one of power

*The final decision of the question must rest with the society, where every member in good standing is entitled to a vote, the president alone excepted.

under the constitution, and not a question of order as to the course of proceeding, which could only be raised by a member of the Association in one of its meetings.

"In my opinion a fair construction of Article 4 of the Association's constitution requires regular meetings of the Association to be held annually. While Section 2 of that Article authorizes the officers, for extraordinary reasons, to change the time and place of meeting upon the written consent of ten of the fifteen officers, I do not think it contemplated a repeal of the first section, which requires annual meetings to be held, but was intended rather to authorize a change of the time and place of holding the annual meeting. My conclusion is that the officers would not be authorized under the constitution to dispense altogether with the annual meeting.

"Yours truly,
"LYMAN TRUMBULL."

With the opinion of a gentleman of such large experience in, and accurate knowledge of, parliamentary proceedings, and acknowledged standing as an able constitutional lawyer, I must accept his interpretation, and decide that the officers of the Association have no power to omit an annual meeting, and therefore direct that the vote just taken be not recorded.

Very truly yours,
W. W. ALPORT,
President.

That the meeting must be held then, would seem to be a settled fact. The only question is as to the time and place, and upon this point the officers no doubt desire the opinion of members. It has been proposed that the meeting should be held in the neighborhood of Washington the week before the meeting of the Medical Congress. This is impracticable, because the Southern Dental Association meets that week, at Old Point Comfort, and it would be a marked discourtesy to that society to select the same time. If the A. D. A. held its meeting the week preceding that, it would imply the spending of three weeks in a hot climate at the most uncomfortable time of the year.

It seems to us that the proper and dignified course for the society is to hold its meeting at or about the usual date, and quite irrespective of any other meeting. This will allow for sufficient time between that and the meeting of the Congress for dentists to return home and prepare for the second great meeting. We are well aware that difficulties attend upon any solution of the problem, but the usual course and time seems to us fraught with less danger than any other, and will reflect greater credit upon the society.

Concerning the place, there is room for diversity of opinion. That the meeting should be called at some other point than Asheville is, we think, conceded on all sides. It should be held at some central and convenient location, easy of access, and where there could be no suspicion of favoritism in its selection. Niagara has always been the compromise place whenever any controversy has arisen during the whole history of the Association, and the wisdom of the choice has never been questioned. It would now, we think, secure a better attendance than any other location, though there are other points which are quite unobjectionable.

As the A. D. A. must uphold the status of dentistry when the Congress shall have departed, let the meeting be held as usual, and the dignity of American dentistry be maintained by the regular course of its onward march, quite irrespective of everything else. Any other plan of procedure will not only be injurious to the A. D. A., but will prejudice the welfare of the Congress, whose success we all are anxious to secure.

In any case, whatever may be the decision of the Executive Committee, it is the duty of every member to accept that decision loyally and without question, and to do all that is possible to secure a meeting memorable for its results, if not for its numbers.

